# Clozapine: prescribing practices in French psychiatric hospitals, multicenter survey on a given day

**DOI:** 10.1192/j.eurpsy.2025.715

**Published:** 2025-08-26

**Authors:** D. Espeillac, E. Queuille, R. Klein

**Affiliations:** 1 Haute-Garonne, Ferrepsy, Toulouse; 2 Gironde, CH Charles Perrens, Bordeaux; 3 Nord, Association du réseau PIC, Armentieres, France

## Abstract

**Introduction:**

Efficacy of clozapine has now been proven for all symptoms of resistant schizophrenia. Yet it remains underused in view of the prevalence of drug resistance and unevenly prescribed worldwide despite a general trend towards increasing prescribing (Bachmann et al. Acta Psychiatr Scand 2017; 1-15). Data on hospital clozapine prescribing in France are older and single-center (Mercier et al. L’Encéphale 2009; 35, 321-329). Collaboration between a national multi-professional network (pharmacist, general practitioner, psychiatrist) working in various public or private mental health establishments (the PIC network) and a regional psychiatric research federation (FERREPSY Occitanie) has enabled a broad and up-to-date study of practices.

**Objectives:**

To assess the prevalence of clozapine prescribing among patients hospitalised in full-time psychiatry on a given day. To assess the prevalence of clozapine prescribing in inpatients with a diagnosis of non-organic psychotic disorder (ICD 10: F20-F29). To study the characteristics of patients treated, prescribing methods and clinical monitoring.

**Methods:**

A cross-sectional observational study was carried out in December 2023 with teams from establishments belonging to the PIC and/or FERREPSY network who had volunteered.

**Results:**

30 centers took part in the study, with a total of 795 patients included. The average age was 44.1 years (66% men and 34% women). 14.05% of hospitalised patients were receiving clozapine treatment on the day of the survey. 25.07% of patients with a diagnosis of non-organic psychotic disorder were receiving clozapine treatment. 26.83% of clozapine prescriptions were off-label, mainly for patients with mood disorders. 91.94% of patients had had their blood pressure measured in the quarter preceding the survey, and 91.82% had been weighed. Conversely, only 31.94% had their umbilical circumference measured.

**Conclusions:**

This study found that the prevalence of prescribing among patients with non-organic psychotic disorders in the hospital was higher than expected, according to European data on clozapine prescription Further data on outpatient use are still required.

**Disclosure of Interest:**

None Declared

